# Hepatic Steatosis Assessment as a New Strategy for the Metabolic and Nutritional Management of Duchenne Muscular Dystrophy

**DOI:** 10.3390/nu14040727

**Published:** 2022-02-09

**Authors:** Ya-Chun Tang, Po-Hsiang Tsui, Chiao-Yin Wang, Yin-Hsiu Chien, Hui-Ling Weng, Chung-Yi Yang, Wen-Chin Weng

**Affiliations:** 1 Department of Medical Imaging and Intervention, Chang Gung Memorial Hospital at Linkou, Taoyuan 333423, Taiwan; tyj@cgmh.org.tw; 2 Department of Medical Imaging and Radiological Sciences, College of Medicine, Chang Gung University, Taoyuan 333323, Taiwan; tsuiph@mail.cgu.edu.tw (P.-H.T.); everybady123@gmail.com (C.-Y.W.); 3 Division of Pediatric Gastroenterology, Department of Pediatrics, Chang Gung Memorial Hospital at Linkou, Taoyuan 333423, Taiwan; 4 Department of Pediatrics, National Taiwan University Hospital, Taipei 100225, Taiwan; chienyh@ntu.edu.tw; 5 Department of Pediatrics, College of Medicine, National Taiwan University, Taipei 100233, Taiwan; 6 Department of Medical Genetics, National Taiwan University Hospital, Taipei 100225, Taiwan; 7 Department of Dietetics, National Taiwan University Hospital, Taipei 100225, Taiwan; jennyweng@ntuh.gov.tw; 8 School of Nursing, College of Nursing, National Taipei University of Nursing and Health Sciences, Taipei 112303, Taiwan; 9 School of Medicine, College of Medicine, I-Shou University, Kaohsiung 824005, Taiwan; cyyang@ntu.edu.tw; 10 Department of Medical Imaging, E-Da Hospital, Kaohsiung 824410, Taiwan; 11 Department of Pediatric Neurology, National Taiwan University Children’s Hospital, Taipei 100226, Taiwan

**Keywords:** duchenne muscular dystrophy, hepatic steatosis, ultrasound imaging, metabolic syndrome

## Abstract

Growing evidence suggests that patients with Duchenne muscular dystrophy (DMD) have an increased risk of obesity and metabolic syndrome (MetS). The aim of this study was to investigate the potential risk factors for MetS and hepatic steatosis in patients with different stages of DMD. A total of 48 patients with DMD were enrolled and classified into three stages according to ambulatory status. Body mass index (BMI), serum fasting glucose, insulin, and lipid profiles including triglycerides (TG) and high-density lipoprotein were measured, and the homeostatic model assessment for insulin resistance (HOMA-IR) index was evaluated. Ultrasound examinations of the liver were performed to assess hepatic steatosis using the Nakagami parameter index (NPI). The results showed that BMI, TG, HOMA-IR, and ultrasound NPI differed significantly among DMD stages (*p* < 0.05). In contrast to the low rates of conventional MetS indices, including disturbed glucose metabolism (0%), dyslipidemia (14.28%), and insulin resistance (4.76%), a high proportion (40.48%) of the patients had significant hepatic steatosis. The ultrasound NPI increased with DMD progression, and two thirds of the non-ambulatory patients had moderate to severe hepatic steatosis. Steroid treatment was a risk factor for hepatic steatosis in ambulatory patients (*p* < 0.05). We recommend that DMD patients should undergo ultrasound evaluations for hepatic steatosis for better metabolic and nutritional management.

## 1. Introduction

Duchenne muscular dystrophy (DMD) is an X-linked neuromuscular disorder with an estimated prevalence of 1–1.5 in 10,000 live-born males [1,2]. It is caused by gene sequence variations in the *dystrophin* gene, resulting in a deficiency of the cytoskeletal protein dystrophin [3]. The characteristic feature of DMD is progressive weakness, mainly involving skeletal muscles, including respiratory muscles, and patients inevitably develop loss of ambulatory ability and respiratory deterioration [4]. In addition, cardiomyopathy and gastrointestinal complications, including dysphagia due to the involvement of cardiac and smooth muscles, also occur with the progression of DMD. If untreated, most patients experience life-threatening events from respiratory or cardiac complications by their early teens [4,5]. Over the past few decades, standardized multidisciplinary care, including the use of glucocorticoids and preventive and appropriate treatment of pulmonary infections, as well as improvements in ventilator care, cardiac management, and spinal surgery, have prolonged ambulation in boys with DMD, delayed pulmonary and cardiac decline, and allowed them to survive into adulthood [6]. Consequently, the picture of DMD patients has changed with the increasing recognition and emphasis on non-motor complications related to prolonged life and treatment-related adverse events [2].

Steroid therapy with prednisone or deflazacort has been proven to slow the progression of muscle degeneration in DMD [2,7]. However, steroid therapy not only affects appearance but also causes adverse effects, including weight gain, osteoporosis, behavioral problems, short stature, and reflux [8,9]. This may at least partially explain why patients in the early stage of DMD experience excessive weight gain or central obesity [10]. Obesity is a risk factor for metabolic syndrome (MetS) and the subsequent development of type 2 diabetes and cardiovascular disease (CVD) [11,12]. Weber et al. reported that glucocorticoid-induced obesity can progress to MetS in patients with DMD, and, therefore, that preventing obesity and evaluating MetS are important for their long-term management [13]. In addition, Rodríguez-Cruz et al. reported that the occurrence of obesity, hyperinsulinemia and insulin resistance (IR) in DMD patients was independent of steroid treatment [14]. Moreover, they also observed a high prevalence of hypertriglyceridemia in patients with DMD and that DMD patients are at risk of MetS, possibly due to increased body fat [15]. For these reasons, the influence of MetS in DMD patients should be taken into consideration to improve health management.

In general, the features that are used to define MetS include disturbed glucose metabolism, arterial hypertension, dyslipidemia, and abdominal obesity [16]. In addition, the homeostatic model assessment for insulin resistance (HOMA-IR) index, calculated using fasting insulin and blood glucose, is also a widely used indicator in clinical research for general evaluations of IR and MetS [17]. Interestingly, the HOMA-IR index has been correlated with sonographic findings of hepatic steatosis [18,19] because nonalcoholic fatty liver is considered to be a hepatic manifestation of MetS [20,21]. A previous case report revealed biopsy-proven hepatic steatosis in a DMD patient, further suggesting an association between DMD and hepatic steatosis [22]. However, the association between DMD and hepatic steatosis has not been validated in large clinical studies. In addition, the significance of hepatic steatosis in the health management of DMD compared to conventional MetS evaluations is also unknown. We hypothesized that patients with DMD may have both hepatic steatosis and MetS. Therefore, the aim of this prospective cohort study was to investigate MetS through anthropometric and biochemical analysis and hepatic steatosis using quantitative abdominal ultrasound in patients with DMD. We also analyzed the potential risk factors for hepatic steatosis in these patients.

## 2. Materials and Methods

### 2.1. Participants

This prospective cohort study was approved by the Institutional Review Board of National Taiwan University Hospital (NTUH). All participants and their legal representatives signed informed consent forms. The study was conducted in accordance with the World Medical Association International Code of Medical Ethics (Declaration of Helsinki). The participants were enrolled from the joint clinics of neuromuscular disorders in the Department of Pediatrics, NTUH. All DMD patients had clinical presentations consistent with DMD and were diagnosed according to muscle biopsies with absent dystrophin and/or the molecular confirmation of *dystrophin* variants. According to effective size = 0.4 and β/α = 1, we enrolled 42 patients that can achieve a power of 0.82.The patients were classified into three stages based on the severity of DMD: stage 1, ambulatory state (patients who were able to walk but with weakness or gait abnormalities); stage 2, early non-ambulatory stage (patients who were unable to walk but able to self-propel or maintain posture); and stage 3, late non-ambulatory stage (patients whose upper limb function and postural maintenance abilities were increasingly limited) based on the DMD CCWG steering committee’s guidelines [2,7]. A pediatric neurologist and a pediatric physiatrist, both with more than 20 years of experience, made the classification.

### 2.2. Anthropometric and Biochemical Analysis

For each patient, clinical information, including age, height, weight, body mass index (BMI), and steroid treatment status, was recorded. For patients who could stand, height was measured using both a wall-mounted stadiometer and point to point (index finger, elbow, shoulder, and across midline) span. For patients unable to stand, height was calculated as a point to point span. The subjects’ weight was calculated wearing light clothing and without shoes with a digital seated scales system. BMI was calculated as weight/height^2^. Serum fasting glucose (mg/dL), insulin (µU/mL), and lipid profiles, including triglycerides (TG) (mg/dL) and high-density lipoprotein (HDL) (mg/dL), were measured from blood samples after 8 h of overnight fasting. Using fasting glucose and insulin data, the HOMA-IR index was then calculated [17].

### 2.3. Ultrasound Examination for Hepatic Steatosis Assessment

All of the enrolled patients underwent abdominal ultrasound examinations by a radiologist who was blinded to the participants’ biochemical data. A clinical ultrasound scanner (Model 3000; Terason, Burlington, MA, USA) equipped with a convex array transducer (Model 5C2A, Terason) was used. The transducer central frequency was 3.5 MHz (bandwidth: 2–5 MHz), and the pulse length was 2.3 mm. An intercostal scanning approach was used to image the liver parenchyma (liver segment VIII) because it reduces the effects of subcutaneous tissues and intestinal gas and provides high stability during liver scanning as the ribs can provide suitable support [23]. For each subject, three effective scans that excluded major vessels and acoustic shadowing were made. The imaging focus and depth were set at 4 and 8 cm, respectively. The raw image data for each scan consisting of 256 scan lines of beamformed radiofrequency (RF) signals were acquired in a breath-hold during gentle normal respiration and stored in an ASCII file.

To evaluate hepatic steatosis, we used the US Food and Drug Administration (FDA)-approved medical software AmCAD-US (AmCad BioMed Corp., Taipei, Taiwan) for offline data analysis of the ASCII files. AmCAD-US allows ultrasound B-mode image reconstruction and generation of Nakagami parametric images, which visually quantifies the echo amplitude distribution using the Nakagami parameter index (NPI) [24,25]. AmCAD-US displays ultrasound B-mode imaging as log-compressed envelopes of the raw image data. Nakagami imaging was constructed by performing sliding window processing of the uncompressed envelope images to calculate local NPI values (the side length of the window was 6.9 mm, and the window overlapping ratio was 95%). The NPI was used as a measure of hepatic steatosis and was obtained by manually outlining the region of interest (ROI) on the Nakagami image to average the pixel values. The echo amplitude distribution has been shown to vary from pre-Rayleigh (NPI < 1) to Rayleigh distribution (NPI = 1) with an increasing severity of hepatic steatosis [24].

### 2.4. Diagnostic Criteria for Metabolic Risk Factors

The diagnostic criteria for metabolic risk factors in adults are not applicable to children and adolescents, and there is currently no international consensus about the definition of MetS in children and adolescents [26]. In this cohort study, we used the following cut-off values commonly used in previous studies as the diagnostic criteria to identify the risks of MetS and hepatic steatosis: BMI for age ≥ 85th and <95th percentile or ≥95th percentile was defined as overweight or obesity, respectively [27]; fasting glucose ≥ 110 mg/dL was defined as disturbed glucose metabolism [28]; TG ≥ 150 mg/dL or HDL < 35 mg/dL was defined as dyslipidemia [28]; HOMA-IR > 3.16 was defined as IR [18]; and NPI > 0.73 was defined as >33% hepatic steatosis (i.e., moderate to severe hepatic steatosis) [24].

### 2.5. Statistical Analysis

Continuous variables were presented as the mean ± standard deviation (SD). Kruskal–Wallis One-way analysis of variance (ANOVA) was performed to compare the difference in each index between different DMD stages. The numbers and percentages of the subjects who fulfilled the metabolic risk criteria for each index were listed and calculated. The data obtained without and with steroid treatment were also compared using the Mann–Whitney U test. A significant difference was defined as a *p* value < 0.05. All statistical analyses were conducted using SigmaPlot 12 (Systat Software, Inc., Richmond, CA, USA).

## 3. Results

### 3.1. Participant Recruitment and Patient Demographics

**Figure 1:**
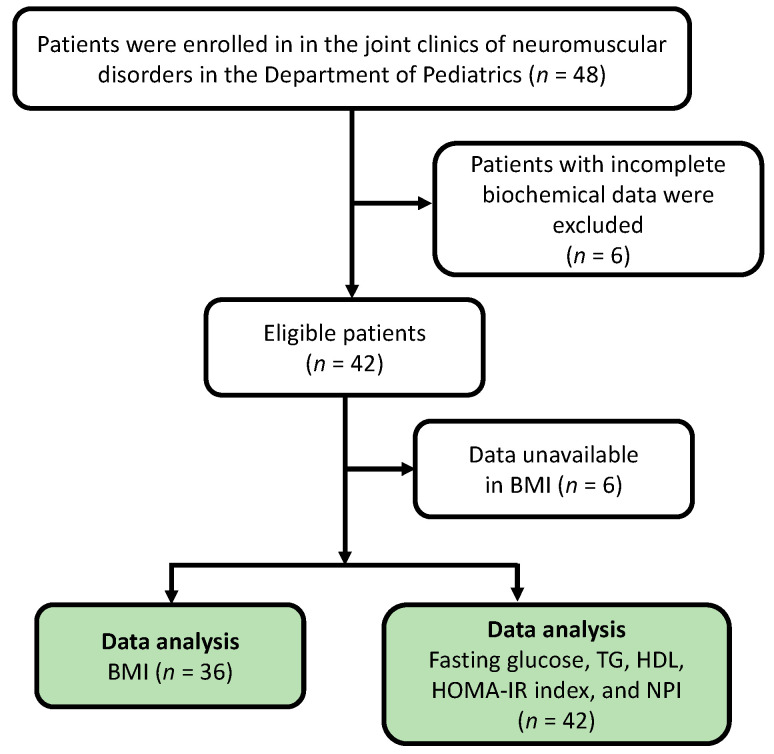
Study enrollment chart. A total of 42 patients met the inclusion criteria and underwent all examinations. The BMI values of six patients were unavailable because of difficulty in measuring their height due to multiple joint contractures. BMI: body mass index; TG: triglycerides; HDL: high-density lipoprotein; HOMA-IR: homeostatic model assessment for insulin resistance; NPI: Nakagami parameter index.

A total of 48 patients (mean (SD) age, 11.93 (6.07) years) were recruited for the study. We excluded six patients who had incomplete biochemical data for analysis. The enrollment chart is illustrated in Figure 1. Of the 42 eligible patients, 21 were in stage 1, 14 were in stage 2, and seven were in stage 3. The patient characteristics, including their clinical symptoms, disease stage, age, and number of patients using steroids, are summarized in Table 1.

### 3.2. Anthropometry and Biochemical Parameters

Table 2 shows the anthropometric and biochemical parameters of the DMD patients at different ambulatory stages. The BMI value in the ambulatory group (stage 1) was 18.87 ± 3.58 kg/m^2^, which increased to 23.39 ± 5.30 kg/m^2^ as the ambulatory ability declined from ambulatory to early non-ambulatory (stage 2). Moreover, for non-ambulatory patients, the BMI decreased significantly when the ambulatory status declined from early non-ambulatory (stage 2) to late non-ambulatory (stage 3) (*p* < 0.05) (Table 2). No significant differences were observed in fasting serum glucose concentration between the three stages of DMD. However, the HOMA-IR index increased from 1.23 ± 0.91 in the ambulatory group (stage 1) to 2.47 ± 2.21 in the early non-ambulatory group (stage 2). In addition, the HOMA-IR index decreased significantly from 2.47 ± 2.21 in the early non-ambulatory group to 0.79 ± 0.26 in the late non-ambulatory group (stage 3) (*p* < 0.05). Notably, the serum TG concentration exhibited a monotonically decrease from 130.66 ± 42.65 mg/dL to 66.85 ± 24.01 mg/dL with declining ambulatory status from stage 1 to stage 3 (*p* < 0.05). However, there was no significant difference in serum HDL concentration between the three ambulatory stages (Table 2).

### 3.3. Hepatic Steatosis Assessment

**Figure 2:**
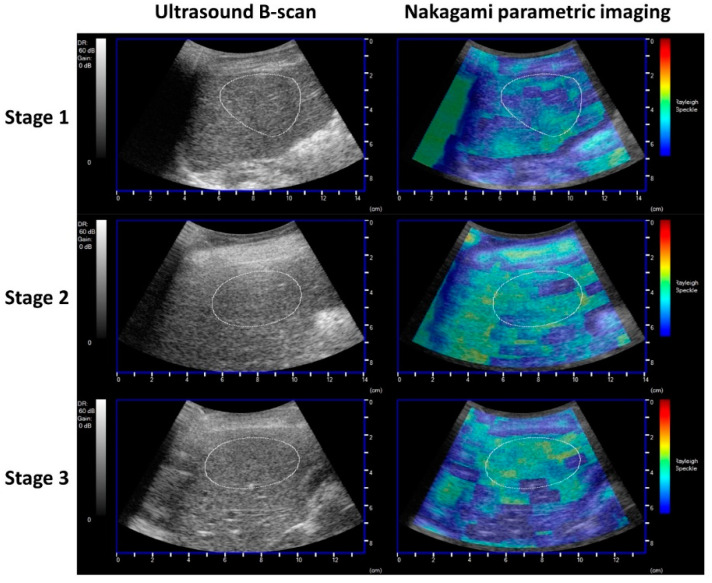
Ultrasound Grayscale B-mode and Nakagami parametric images of the liver in DMD patients at different ambulatory stages (stage 1: ambulatory stage, stage 2: early non-ambulatory stage, stage 3: late non-ambulatory stage). The brightness of ultrasound Nakagami imaging gradually increased with declining ambulatory stage of DMD. The NPI as a measure of hepatic steatosis was obtained by manually outlining the region of interest (ROI) (dotted line) on the Nakagami image for averaging the pixel values.

Figure 2 shows ultrasound grayscale B-mode and Nakagami images of the liver at each ambulatory stage of DMD, as constructed using AmCAD-US. The brightness of ultrasound Nakagami imaging gradually increased with declining ambulatory status, meaning that the echo amplitude distribution varied toward the Rayleigh distribution. The NPI value of the ROI on the Nakagami images, a measure of hepatic steatosis, increased significantly from 0.61 ± 0.11 to 0.74 ± 0.07 as the ambulatory ability declined from ambulatory to non-ambulatory (*p* < 0.05). In addition, the NPI value of the patients in the late non-ambulatory stage was similar to that of the patients in the early non-ambulatory stage, implying persistent hepatic steatosis in the late non-ambulatory group with undernutrition and decreased HOMA-IR index (Table 2).

### 3.4. Metabolic Risks

Table 3 shows the prevalence of each parameter of metabolic risk and hepatic steatosis in our DMD cohort. Overall, 52.38% of the ambulatory patients (stage 1) had overweight or obesity, 28.57% had dyslipidemia with a high serum TG concentration, and 14.28% had moderate to severe hepatic steatosis. Disturbed glucose metabolism and IR were not found in the ambulatory patients. In comparison, 33.33% of the early non-ambulatory patients had overweight or obesity, 14.28% had dyslipidemia with a low serum HDL concentration, and 14.28% had IR, while none of the late non-ambulatory patients fulfilled the criteria for any of the metabolic risks. None of the non-ambulatory patients fulfilled the criteria for disturbed glucose metabolism or dyslipidemia with a high serum TG concentration. Of note, 14 of the 21 (66.67%) non-ambulatory DMD patients had moderate to severe hepatic steatosis.

### 3.5. The Role of Steroid Treatment in Metabolic Risks and Hepatic Steatosis

Table 4 shows comparisons of anthropometric and biochemical parameters between the groups without and with steroid treatment. There were no significant differences in values of BMI, fasting serum glucose, serum TG concentration, serum HDL concentration, and HOMA-IR between the DMD patients who did and did not receive steroid treatment regardless of the ambulatory status. However, the NPI value in the ambulatory subjects who received steroid treatment was significantly higher than that in those who did not receive steroid treatment (0.63 ± 0.10 versus 0.52 ± 0.06, *p* < 0.05).

## 4. Discussion

In this study, we investigated MetS and hepatic steatosis in patients with DMD with the aim of contributing evidence-based insights to clarify issues related to the metabolism of DMD patients. We found significant differences in BMI, serum TG concentration, HOMA-IR index, and ultrasound NPI between different ambulatory stages of DMD, representing that patients with DMD present with varying degrees of obesity, impaired glucose metabolism, dyslipidemia, and hepatic steatosis with the decline in ambulatory ability. Although the prevalence rates of individual components of MetS, including disturbed glucose metabolism, dyslipidemia, and IR, were low in our patients, a surprising proportion had significant hepatic steatosis, the hepatic component of MetS, especially in the non-ambulatory patients and regardless of obesity. To the best of our knowledge, this is the first study to investigate hepatic steatosis in patients with DMD.

In general, patients at the early stage of DMD have an increased risk of obesity due to reduced physical activity, decreased caloric requirements, and increased appetite caused by steroid therapy, leading to excessive caloric intake [29]. Our results also support that patients with DMD are at an increased risk of being overweight or obese not only during the ambulatory stage but also during the early non-ambulatory stage. Importantly, our results show that patients usually transition from obesity to malnutrition with a significant decrease in BMI as the disease progresses to an advanced stage. This may be related to increased energy demand due to respiratory failure and difficulties in swallowing and food intake because of weakened oropharyngeal muscles [30,31,32,33]. Of note, an obvious decrease in serum TG level was found in our non-ambulatory patients with DMD, which represents the presence of malnutrition or chronic disease [34]. Interesting, our patients had a lower percentage of IR compared to previous studies [14]. In our hospital, we provide integrated multidisciplinary care, including nutritional management, for our patients with DMD. This may be the reason why our patients had lower rates of IR and other biochemical components of MetS. Our results also support the importance of standardized multidisciplinary management for patients with DMD to improve their MetS [2].

Hepatic steatosis is the hepatic manifestation of MetS. Compared with the low rates of conventional MetS indices, the high percentage of hepatic steatosis in our cohort suggests that hepatic steatosis may be clinically more important and meaningful when evaluating and managing patients with DMD. Our results revealed that hepatics steatosis was very common in the non-ambulatory patients, including those in wheelchairs and those who were bedridden, regardless of whether or not they were obese. A number of potential mechanisms can contribute to hepatic steatosis in DMD patients. Previous body composition studies have shown that, compared with healthy controls, non-obese DMD patients had higher fat mass, especially those who had lost walking ability [14,35]. This may explain the high proportion of hepatic steatosis in our non-ambulatory patients. In addition, in our patients with preserved walking ability, steroid therapy was a risk factor for hepatic steatosis. Steroids can stimulate lipid deposition pathways to increase the release of free fatty acids from adipose stores, which are then taken up by the liver leading to hepatic steatosis [36]. In our cohort, we found that the rate of steroid use in patients after loss of ambulation significantly decreased, and the non-ambulatory patients who continued to use steroids also received much lower doses than ambulatory patients (data not shown) due to benefit-risk-ratio considerations at this stage, whereas the adverse effects of steroid use often outweigh the benefits. Therefore, steroid treatment may be an important risk factor for developing MetS, especially hepatic steatosis, in ambulatory patients but not in non-ambulatory patients. In non-ambulatory patients, malnutrition, not steroid treatment, leading to severe impairments of hepatic peroxisomal and mitochondrial function may be a potentially important mechanism for hepatic metabolic dysfunction [37]. Furthermore, protein deficiency can also lead to a reduction in plasma TG and phospholipids, an increase in free fatty acids, and hepatic steatosis, which is associated with the accumulation of hepatic TG due to a reduction in hepatic phospholipids [38]. Taken together, these findings suggest that hepatic steatosis could be present in DMD patients with malnutrition, and that fatty liver detection should be considered when performing nutritional management in DMD patients.

As shown in our current and recent studies, complications resulting from malnutrition are frequently seen in DMD patients [2]. Advances in DMD management have resulted in a longer life expectancy; thus, nutritional issues and complications related to adulthood should be considered. Hence, it is necessary to revise the existing management of complications and standard of care in DMD and pay more attention to adult care to improve the quality of life of DMD survivors [39]. Based on our findings, we strongly recommend that DMD patients should be assessed for hepatic steatosis using ultrasound imaging as a quick screening tool. Liver biopsy is the gold standard to diagnose hepatic steatosis; however, it cannot be routinely used due to its invasiveness and sampling errors. Noninvasive imaging techniques are suitable clinically as alternative assessment tools for hepatic steatosis. In particular, ultrasound is a cost-effective and widely available tool which can provide real-time results without the use of ionizing radiation. Moreover, DMD patients usually transition from obesity to malnutrition at a later stage, while the biochemical analysis of MetS may return to the normal range and does not truly reflect their nutritional and metabolic abnormalities. At this stage, the examination of hepatic steatosis will be more important and relevant than biochemical analysis of MetS to reflect the nutritional and metabolic problems of patients.

Currently, the controlled attenuation parameter (CAP) based on vibration-controlled transient elastography (Fibroscan, Echosens, Paris, France) is the most frequently used clinical tool to evaluate hepatic steatosis [40]. However, the major drawback of Fibroscan is that it does not provide direct image guidance during the examination [41], limiting its use for general standard-care abdominal ultrasound examinations in DMD patients. This is also the reason why we chose NPI as the quantitative analysis method for hepatic steatosis. Ultrasound NPI calculated using AmCAD-US is a new FDA-approved technique that can be used to assess hepatic steatosis with a general ultrasound imaging system. Ultrasound NPI provides a strong link between physical meaning to changes in the microstructures of the liver parenchyma due to hepatic steatosis [24]. A standard-care abdominal ultrasound examination integrated with quantitative analysis using AmCAD-US may have great potential in hepatic steatosis detection and health management of patients with DMD. Moreover, considering that hepatic steatosis is a risk factor for steatohepatitis, evaluating liver fibrosis in DMD may be an interesting issue in the future.

This study has some limitations. First, due to the rareness of DMD, the number of patients enrolled in this study was relatively limited. Second, no histological evidence of hepatic steatosis was provided due to the difficulties in performing liver biopsies in the DMD patients. However, sonographic findings confirmed by quantitative ultrasound are promising as an alternative assessment of hepatic steatosis in DMD. Third, this study was a cross-sectional design, not a longitudinal investigation. Further longitudinal studies are warranted to investigate the longitudinal changes of MetS, including hepatic steatosis, in DMD survivors and the effects of hepatic steatosis on steroid treatment planning and nutritional management.

## 5. Conclusions

In conclusion, in this cohort study, we explored MetS and hepatic steatosis in patients with DMD using quantitative ultrasound, as well as changes in metabolic risk factors. BMI, serum TG concentration, and HOMA-IR index were found to differ significantly between different stages of DMD. Compared with the low rates of conventional MetS indices, including disturbed glucose metabolism, dyslipidemia, and IR, in our patients, our novel approach using ultrasound NPI showed a high rate of significant hepatic steatosis, implying that ultrasound NPI may be a more relevant imaging biomarker to monitor metabolic alterations in patients with DMD. In patients with preserved walking ability, the use of steroids may be a risk factor for the formation of hepatic steatosis to some degree. In non-ambulatory patients, the ultrasound NPI increased with DMD progression, which may be related to the effect of malnutrition. Taken together, hepatic steatosis is a nonnegligible issue when considering the metabolic and nutritional management of patients with DMD, and we recommend that it should be integrated into the standard of care for DMD survivors. A combination of anthropometric, biochemical, and ultrasound analyses for evaluating MetS and hepatic steatosis is recommended to more comprehensively assess nutritional and metabolic issues and to enable better management in DMD patients.

## Figures and Tables

**Table 1:** The demographic data of the DMD patients at different ambulatory stages.

Stage	Clinical Symptoms	Age (Years) Median (Range)	No. of Subjects (With Steroid Use)
Stage 1	Early ambulatory: Showing a Gowers’ sign (patients need to support themselves with hands to get up from the floor), waddling type walking (gait), and walking on their toes. Late ambulatory: Walking becomes increasingly difficult (labored gait) and climbing stairs and getting up from the floor are more problematic.	8.5 (3–18.5)	21 (16)
Stage 2	Early non-ambulatory: Patients start to need to use a wheelchair; they may be able to wheel the chair themselves and typically their postures can be maintained even scoliosis is possible.	15.5 (8.9–18.3)	14 (9)
Stage 3	Late non-ambulatory: Upper limb function and maintenance of good posture are increasingly difficult, and complications are more likely.	18.1(14.1–26.2)	7 (1)

Data are presented as mean ± SD. Stage 1: ambulatory subjects. Stages 2 and 3: non-ambulatory subjects.

**Table 2:** Anthropometric and biochemical parameters of the DMD patients at different ambulatory stages.

Parameters	Stage 1 Ambulatory (*n* = 21)	Stage 2 Early Non-Ambulatory (*n* = 14)	Stage 3 Late Non-Ambulatory (*n* = 7)	*p* Value
BMI (kg/m^2^)	18.87 ± 3.58	23.39 ± 5.30 ^1^	14.94 ± 4.29 **	0.02
Fasting glucose (mg/dL)	86.66 ± 7.15	85.14 ± 9.44	83.57 ± 6.18	0.64
TG (mg/dL)	130.66 ± 42.65 **^,1^	86.07 ± 20.87 *	66.85 ± 24.01 *	<0.001
HDL (mg/dL)	53.76 ± 10.95	50.07 ± 11.75	48.71 ± 9.74	0.46
HOMA-IR	1.23 ± 0.91	2.47 ± 2.21 ^1^	0.79 ± 0.26 **	0.006
NPI	0.61 ± 0.11 **^,1^	0.74 ± 0.07 *	0.74 ± 0.08 *	<0.001

Data are presented as mean ± SD. The statistic power is 0.82. *: *p* < 0.05 compared with the stage 1. **: *p* < 0.05 compared with the stage 2. ^1^: *p* < 0.05 compared with the stage 3. BMI: body mass index; TG: triglycerides; HDL: high-density lipoprotein; HOMA-IR: homeostatic model assessment for insulin resistance; NPI: Nakagami parameter index.

**Table 3:** The percentage of each parameter of metabolic risk and hepatic steatosis in our DMD cohort. Two thirds of the non-ambulatory DMD patients had moderate to severe hepatic steatosis.

	No. of Subjects Who Fulfill the Criteria/No. of Subjects (%)	No. of Subjects Who Fulfill the Criteria/No. of Ambulatory Subjects (Stage 1) (%)	No. of Subjects Who Fulfill the Criteria/No. of Subjects at Stage 2 (%)	No. of Subjects Who Fulfill the Criteria/No. of Subjects at Stage 3 (%)
BMI ≥ 85th percentile	16/42 (38.09%)	12/21 (52.38%)	4/12 (33.33%)	0/3 (0%)
Fasting glucose ≥ 110 (mg/dL)	0/42 (0%)	0/21 (0%)	0/14 (0%)	0/7 (0%)
TG ≥ 150 (mg/dL)	6/42 (14.28%)	6/21 (28.57%)	0/14 (0%)	0/7 (0%)
HDL < 35 (mg/dL)	2/42 (4.76%)	0/21 (0%),	2/14 (14.28%)	0/7 (0%)
HOMA-IR > 3.16	2/42 (4.76%)	0/21 (0%)	2/14 (14.28%)	0/7 (0%)
NPI > 0.73	17/42 (40.48%)	3/21 (14.28%)	10/14 (71.43%)	4/7 (57.14%)

Data are presented as mean ± SD. BMI ≥ 85th percentile: overweight or obesity; Fasting glucose ≥ 110 mg/dL: disturbed glucose metabolism, TG ≥ 150 mg/dL or HDL < 35 mg/dL: dyslipidemia; HOMA-IR > 3.16: insulin resistance; NPI > 0.73: hepatic steatosis > 33%.

**Table 4:** Comparisons of anthropometric and biochemical parameters between the groups without and with steroid treatment. The significant differences were found in NPI of ambulatory patients.

	Without Steroid Treatment	With Steroid Treatment	*p* Value
Ambulatory subjects (*n* = 21)	5	16	
BMI (kg/m^2^)	16.71 ± 1.11	19.55 ± 3.84	0.24
AC glucose (mg/dL)	88.61 ± 7.98	86.06 ± 7.03	0.60
TG (mg/dL)	130.61 ± 39.81	130.68 ± 44.76	0.78
HDL (mg/dL)	48.20 ± 10.32	55.50 ± 10.86	0.16
HOMA-IR	0.91 ± 0.51	1.34 ± 0.99	0.46
NPI	0.52 ± 0.06	0.63 ± 0.10	0.02 **
Non-ambulatory subjects (*n* = 21)	11	10	
BMI (kg/m^2^)	17.44 ± 4.54	23.94 ± 5.88	0.06
AC glucose (mg/dL)	85.09 ± 5.94	84.10 ± 10.76	0.18
TG (mg/dL)	72.90 ± 22.57	87.10 ± 22.83	0.18
HDL (mg/dL)	47.01 ± 4.95	52.50 ± 14.79	0.13
HOMA-IR	1.35 ± 1.14	2.54 ± 2.51	0.06
NPI	0.74 ± 0.05	0.74 ± 0.08	0.48

Data are presented as mean ± SD. Ambulatory patients: stage 1. Non-ambulatory patients: stages 2–3. **: *p* < 0.05.

## Data Availability

The data presented in this study are available on request from the corresponding author.
